# Molecular and Biochemical Characterization of *Xanthomonas arboricola* pv. *corylina* Isolates Infecting Hazelnut Orchards in Chile

**DOI:** 10.3390/plants14203148

**Published:** 2025-10-13

**Authors:** Gastón Higuera, Brenda Ossa, Alan Zamorano, Pamela Córdova, Belén Díaz, Sebastián Cabrera, Tomás Llantén, Javiera Fuentes, Camila Gamboa, Weier Cui, Assunta Bertaccini, Carolina Ilabaca-Díaz, Set Pérez Fuentealba, Simón Navarrete, Héctor García, Nicola Fiore

**Affiliations:** 1 Instituto de Nutrición y Tecnología de los Alimentos, Universidad de Chile, Avenida El Líbano 5524, Macul, Santiago 7830490, Chile; gastonhiguera@inta.uchile.cl (G.H.); pamela.cordova@inta.uchile.cl (P.C.); belen.diaz@inta.uchile.cl (B.D.); cilabaca@inta.uchile.cl (C.I.-D.); 2 Facultad de Ciencias Agronómicas, Universidad de Chile, Avenida Santa Rosa 11315, La Pintana, Santiago 8820808, Chile; brenda.ossa@ug.uchile.cl (B.O.); agezac@u.uchile.cl (A.Z.); sebastian.cabrera@ug.uchile.cl (S.C.); tomas.llanten@ug.uchile.cl (T.L.); javiera.fuentes.1@ug.uchile.cl (J.F.); camila.gamboa@uchile.cl (C.G.); weier.cui@uchile.cl (W.C.); 3 *Alma Mater Studiorum*-University of Bologna, 40127 Bologna, Italy; 4 Instituto de Ciencias Agroalimentarias, Animales y Ambientales (ICA3), Universidad de O’Higgins, Ruta 90 Km. 3, San Fernando 3070000, Chile; set.perez@uoh.cl; 5 ANASAC Chile S.A., Almirante Pastene 300, Providencia, Santiago 7500506, Chile; snavarrete@anasac.cl; 6 Laboratorios Diagnofruit Ltda, Sucre 1521, Ñuñoa, Santiago 7750109, Chile; hgarcia@diagnofruit.cl

**Keywords:** bacterial blight, MLSA, phylogeny, metabolism

## Abstract

In recent years, the cultivated area of hazelnuts in Chile has increased significantly. Along with this rapid expansion, biotic constraints that affect the optimal development of the crop have been identified. Among these, bacterial blight disease caused by *Xanthomonas arboricola* pv. *corylina* has been particularly relevant. This pathogen has a global distribution and is present in all hazelnut-producing countries. In the spring of 2023, hazelnut orchards were sampled from the Maule to Biobío Regions of Chile. The Chilean isolates recovered from hazelnut tissues showing symptoms of bacterial blight were characterized by their ability to grow on different semi-selective media, their carbohydrate utilization profiles, hypersensitivity response in tobacco plants, and biochemical tests. Additionally, the isolates were identified based on the 16S rRNA gene and multilocus sequence analysis (MLSA) on the *rpoD*, *gyrB,* and *atpD* genes. The results showed that the *X. arboricola* pv. *corylina* Chilean isolates differed from previously reported isolates in other geographic areas as they are capable of metabolizing sorbitol and mannitol. Using MLSA and average nucleotide identity (ANI) comparison, these isolates were grouped into four and five phylogenetic clades, respectively, representing a significant difference from what has been reported in similar international studies.

## 1. Introduction

*Xanthomonas arboricola* is a bacterial species known to cause damage to fruit trees worldwide [1]. This species comprises nine pathovars [2] and among them, *Xanthomonas arboricola* pv. *corylina* has been identified as the causal agent of bacterial blight of hazelnut [3]. This disease was first reported in Oregon, United States of America, and is currently present in all European hazelnut cultivation areas [4,5].

*X. arboricola* pv. *corylina* is a highly specific pathogen that affects only hazelnuts (*Corylus* spp.), causing significant economic losses [6]. Lamichhane and Varvaro [4] report that *X. arboricola* pv. *corylina* reduces plant lifespan as well as the quantity and quality of fruit production. In young plants, mortality rates can reach up to 10%, while in older trees the damage is even more severe, sometimes reaching 100%.

*X. arboricola* pv. *corylina* is an epiphytic bacterium that enters the host through natural openings, leaf wounds, pruning cuts, and frost damage. Its symptoms include wilted shoots during spring and summer, leaves with chlorotic and brown spots, and slightly sunken brown cankers on the bark, visible in spring [4,5]. High rainfall, late spring frosts, soils with elevated nitrogen and low magnesium content, presence of diverse types of stress, high clay content in the soil, and low drainage capacity have been identified as favorable conditions for the spread of the disease, increasing the susceptibility of cultivars to *X. arboricola* pv. *corylina* [3,4,6].

On the other hand, exposure to copper-based products applied to the orchard over the years to control bacterial diseases has been associated with the increasing copper tolerance in several phytopathogenic bacterial isolates In the case of *X. arboricola* pv. *corylina*, differences in copper tolerance were observed depending on the geographic origin, with isolates in Turkey being found with resistance to copper sulfate concentrations of up to 2.56 mM, compared to isolates isolated from Poland and Serbia that showed a resistance of 0.64 and 0.32 mM, respectively, the latter value recorded as the tolerance threshold [6,7]. In this context, the frequency of resistant strains in the population increases over time as the plant is exposed to copper-based chemicals, acquiring resistance by mutation or conjugation [5,6].

Several phylogenetic studies have shown that the structure of the *X. arboricola* pv. *corylina* population has peculiar characteristics that are unrelated to the geographical origin of the isolates [5]. Scortichini et al. [8] point out that there is genetic variability among *X. arboricola* pv. *corylina* isolates; however, the different phylogenetic groups discovered include isolates from different geographical areas, with strains from different continents having the same profile, while others, isolated from the same area, differ. Furthermore, Webber et al. [6] point out that the type strain of *X. arboricola* pv. *corylina* isolated in Oregon differs from isolates from other countries.

In Chile, the hazelnut cultivated area has experienced a considerable increase since the first 12 hectares were planted in 1990 [9]. The planted area extends from the Metropolitan Region to Los Lagos, currently exceeding 36,000 hectares, with the Maule Region having the largest production (more than 45%) [10]. The favorable climatic conditions for the development of the crop in the country and its off-season harvest compared to the northern hemisphere offer a fresh product with optimal organoleptic characteristics for the agroindustry [11]. This growth has caused high demand for plant material in nurseries; however, the crop health condition was not verified or certified [12].

Bacterial blight of hazelnut was first reported in Chile in 1987 at the Carillanca experimental station (INIA, Araucanía Region) in trees from Oregon, United States of America [13], even though the first commercial hectares had not yet been planted. Previously in the country, infections by the bacterium were described in young plants and in nurseries, with an incidence between 60% and 90% [3] and harvest losses of 30% [11]. Previous studies have also described the symptoms of the disease, the phenotypic and genotypic characteristics of the *X. arboricola* pv. *corylina* isolate present in Chile, and the complete genome of a strain has been obtained in the Maule Region [14]. However, to date, few studies have been conducted on the phylogenetic diversity of *X. arboricola* pv. *corylina* isolated from Chilean orchards. In the present study, several *X. arboricola* pv. *corylina* isolates were molecularly and biochemically characterized to contribute to the understanding of the *X. arboricola* pv. *corylina* population features at the local level. This will help to optimize the management of the bacterium. The results revealed distinctive features of the Chilean *X. arboricola* pv. *corylina* isolates in comparison to those reported in other geographic areas, such as the capability of metabolizing sorbitol and mannitol. Also, the Chilean *X. arboricola* pv. *corylina* isolates showed higher diversity, being grouped into four or five phylogenetic clades when analyzed through multilocus sequence analysis (MLSA) or average nucleotide identity (ANI) comparison, respectively, representing a significant difference from what has been reported in previous studies.

## 2. Results

### 2.1. Isolation and Identification of Xanthomonas *spp.*

**Figure 1:**
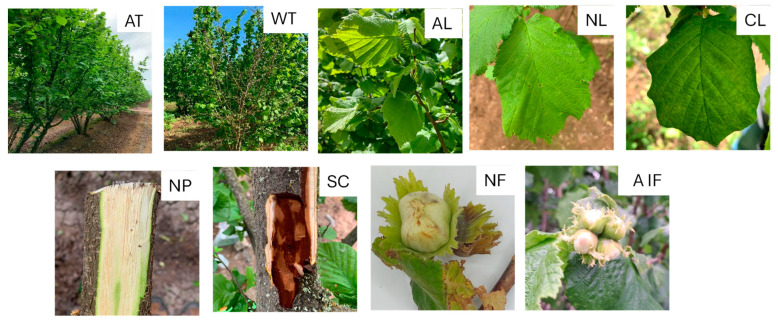
Hazelnut plant material exhibiting different symptoms. AT: asymptomatic trees; WT: wilted twigs; AL: asymptomatic leaves; NL: leaves with necrotic spots; CL: leaves with chlorotic spots; NP: portions of necrotic phloem; SC: stem canker; NF: necrotic fruit; AIF: asymptomatic involucres and fruit.

For bacterial isolation, plant material was collected from European hazelnut trees with different disease symptoms, such as leaf necrotic or chlorotic spots, asymptomatic leaves, dry twigs, cankers, fruit involucres, asymptomatic fruits, and fruits with necrotic lesions (Figure 1).

A total of 49 samples were collected and grouped in 113 subsamples, which were cultured on King’s B medium (KB) [15] for morphological characterization and subsequent selection of bacterial isolates. The initial selection occurred based on the macroscopic characteristics of the colonies, such as color, shape, shine, and mucous-like appearance. Using these criteria, 166 isolates were chosen. Bacterial colonies exhibiting the classical morphology of *Xanthomonas* on KB medium—mucoid, spherical/convex colonies with a defined smooth border, shiny surface, and bright yellow color—were selected for identification by 16S rRNA sequencing after amplification (Figure S1). Analyses of the 16S rRNA gene sequences determined that 18 out of 49 e (36.7%) samples, 13 (26.5%) symptomatic and five (10.2%) asymptomatic, resulted positive. Twenty-nine out of the 160bacterial isolates analyzed correspond to the genus *Xanthomonas* (Table S1). The other isolates belong to different genera, such as *Microbacterium, Bacillus*, *Pseudomonas*, *Pantoea*, *Erwinia*, *Paenarthrobacter*, *Curtobacterium*, *Stenotrophomonas*, and *Micrococcus*, among others.

*Xanthomonas* isolates were obtained from tissue samples of leaves with necrotic spots, chlorotic spots, cankers, fruit involucre and exudate, and also symptomless leaves. A total of five varieties were sampled: Tonda di Giffoni, Barcelona, Lewis, Blanco Azul, and Mixta. However, *Xanthomonas* spp. was successfully isolated only from Tonda di Giffoni and Barcelona.

### 2.2. Molecular Characterization of X. arboricola *pv.* corylina Isolates

To achieve the molecular characterization of the 29 isolates identified as belonging to the *Xanthomonas* genus, their *rpoD* and *gyrB* genes were amplified by PCR.

Using the BLASTn tool of the NCBI (National Center for Biotechnology Information), nucleotide sequence comparisons confirmed that the amplification products corresponded to *X. arboricola* pv. *corylina*. The sequences of the *rpoD* and *gyrB* genes from the 29 bacterial isolates were used for MLSA analyses (Figure S6). Sequences from reference strains of *X. arboricola* pv. *corylina* and the pathovars *juglandis* and *pruni* (Table S2), available in the GenBank database, were included in the analyses.

**Figure 2:**
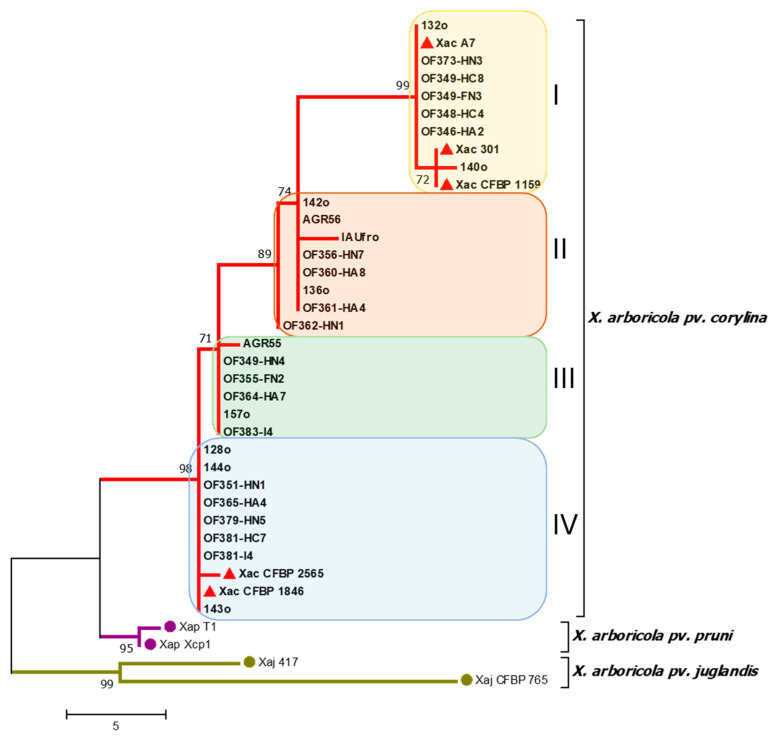
Phylogenetic tree using the Maximum Parsimony method obtained by multilocus analysis (MLSA) combining the nucleotide sequences of the *rpoD*, *gyrB*, and *atpD* genes. Evolutionary distances were calculated using the Composite Maximum Likelihood method. Each color indicates a distinct phylogenetic group of *X. arboricola* pv. *corylina*. The red triangles indicate the *X. arboricola* pv. *corylina* reference strains. Chilean and reference isolates’ GenBank accession numbers are reported in Tables S1 and S2.

The nucleotide sequences obtained for each gene from the different isolates were aligned and trimmed, yielding sequences of 880 and 780 bp in length for the *rpoD* and *gyrB* genes, respectively. Furthermore, to complement the phylogenetic analyses, the sequences of the *atpD* gene of the 29 *X. arboricola* pv. *corylina* whole-genome isolates were included. The gene sequences were aligned and concatenated, ending in a sequence of 3032 bp. An MLSA was performed using the three genes, *rpoD*, *gyrB,* and *atpD* (Figure 2).

With the 29 isolates used for the construction of the phylogenetic tree (Figure 2), four clades were obtained using the Maximum Parsimony method. The four clades obtained were numbered I to IV. The reference bacterial isolates of *X. arboricola* pv. *corylina* were grouped into clades I and IV, with two clades enclosing only the Chilean isolates obtained in the present study.

Regarding the geographic distribution of the phylogenetic groups (Figure S2), the first group (clade I) included isolates present in three localities (Teno, Chillán, and Los Ángeles) in the Maule, Ñuble, and Biobío Regions; the second (clade II) grouped isolates from three localities (Chillán, Coihueco, and Gorbea) in the Ñuble and Araucanía Regions; the third (clade III) grouped isolates present in four localities (Melocotón, Chillán, Coihueco, and Panguipulli) from the Maule, Ñuble, and Los Ríos Regions; the fourth group (clade IV) included isolates present in seven localities (Chillán, Coihueco, Los Ángeles, Coipue, Gorbea, Villarrica, and Pitrufquén) from the Ñuble to Araucanía Regions. This last group covers the largest geographic distribution within the country.

**Figure 3:**
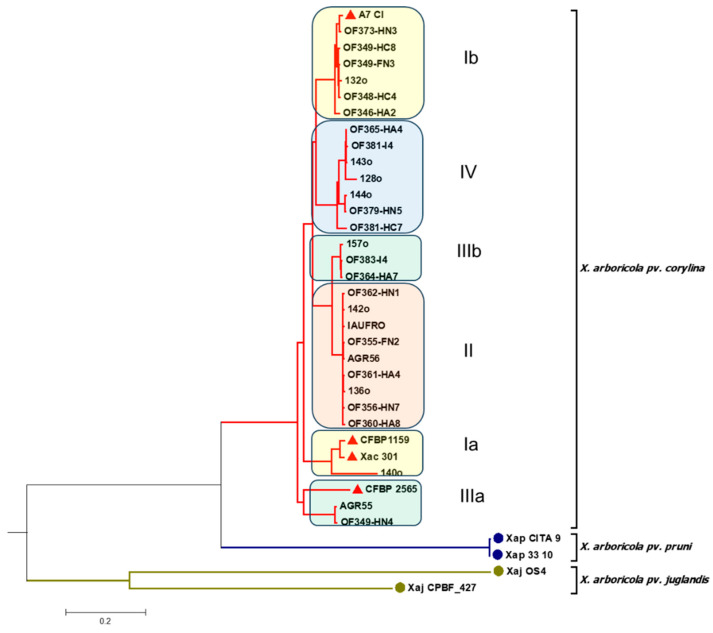
Phylogenetic tree using the average nucleotide identity (ANI) for the genomic sequences of 29 *X. arboricola* pv. *corylina* isolates. The red triangles indicate the *X. arboricola* pv. *corylina* reference isolates. Chilean and reference isolates’ GenBank accession numbers are reported in Tables S1 and S2.

To further analyze the diversity of the 29 *X. arboricola* pv. *corylina* isolates, whole-genome sequencing was used (Figure 3 and Figure S5).

When using the complete genome sequences of the 29 *X. arboricola* pv. *corylina* isolates, six clades were identified. However, when compared with the MLSA-based phylogenetic tree, it could be observed that only 24 of the 29 Chilean isolates cluster as in the MLSA tree. In this context, the isolates *X. arboricola* pv. *corylina* 140o and Xac 301 remain close but are separated from the rest of the *X. arboricola* pv. *corylina* isolates that are part of clade I according to the MLSA tree. On the other hand, from the six isolates that constitute clade III in the MLSA tree, OF364-HA7, 157o, and OF383-I4 group together again, but AGR55 and OF349-HN4 cluster in a different clade (Group IIIa), while OF355-FN2 belongs to group II. Finally, it is noted that, when using the complete genomes, the reference strains *X. arboricola* pv. *corylina* CFBP 1159, *X. arboricola* pv. *corylina* 301, and *X. arboricola* pv. *corylina* CFBP 2565, clustering differs from that observed in the MLSA tree. However, the isolate 140o remains the closest to the *X. arboricola* pv. *corylina* CFBP 1159 and *X. arboricola* pv. *corylina* 301 reference strains.

### 2.3. Biochemical Characterization of Xanthomonas arboricola *pv.* corylina Isolates

The biochemical characterization of the 29 *X. arboricola* pv. *corylina* isolates showed that they responded positively to growth at 35°C, starch hydrolysis, NaCl tolerance (5%), gelatin liquefaction, and growth in media containing carbon sources such as sucrose, mannitol, sorbitol, glucose, and trehalose (Figure S3). On the other hand, all the analyzed strains were negative to the oxidase test (Tables S3 and S4).

### 2.4. Hypersensitivity Response of Xanthomonas arboricola *pv.* corylina Isolates

To determine the pathogenic potential of the *X. arboricola* pv. *corylina* isolates identified by molecular analyses, a hypersensitivity response (HR) assay was performed on tobacco plant leaves. Of the 29, only twenty-five exhibited HR in tobacco leaves 48 h after infiltration. The remaining four isolates caused chlorosis. Neither chlorosis nor HR was observed in tobacco leaves infiltrated with an isolate of *Pantoea agglomerans* (negative control). Meanwhile, a positive response was observed in leaf areas infiltrated with *Pseudomonas syringae* pv. *actinidiae* (positive control) (Figure S4 and Table S5).

## 3. Discussion

In the present study, *X. arboricola* pv. *corylina* isolates were obtained from hazelnut orchards of five regions of Chile, including 12 geographic locations. For *X. arboricola* pv. *corylina* isolation, samples of different symptomatic and asymptomatic tissues were used. It was observed that most of the *X. arboricola* pv. *corylina* isolates were from leaves with necrotic spots; however, the bacteria could also be isolated from leaves with chlorotic spots and asymptomatic leaves of trees that showed symptoms in adjacent shoots. The latter is particularly relevant when considering the importance of early diagnosis and detection of the disease, which may be present in a latent period without presenting symptoms, as evidenced by the results of this survey.

The bacteria could not be isolated from the fruits, which is not surprising since the pathogen rarely affects them [7]. A total of five varieties were sampled: Tonda di Giffoni, Barcelona, Lewis, Blanco Azul, and Mixta. However, *X. arboricola* pv. *corylina* was only isolated from the first two, which are also the most cultivated varieties in Chile. Sampling was carried out on trees planted from 2005 to 2021. *X. arboricola* pv. *corylina* isolates were mainly present in trees planted during 2016 and 2017; however, the bacteria were also found in a tree planted in 2005. These findings suggest that the bacterium is gradually establishing itself in hazelnut orchards over time. This could serve as an explanation for why the varieties that have been in Chile longer may exhibit higher disease incidence.

When assessing the genetic diversity of the 29 *X. arboricola* pv. *corylina* Chilean isolates, phylogenetic trees were constructed based on an MLSA analysis of the *rpoD*, *gyrB,* and *atpD* housekeeping genes (using the Maximum Parsimony method) and also based on the comparison of isolates’ whole-genomic sequences via the ANI approach. Four and five phylogenetic groups (clades) were identified using MLSA analysis and the ANI approach, respectively. This difference is due to the higher resolution of ANI compared to MLSA analysis. The MLSA analysis by Webber et al. [6], concatenating the *rpoD* and *gyrB* genes, shows two *X. arboricola* pv. *corylina* clades separated by different *X. arboricola* pathovars. This was not observed in the present study, for which longer, and therefore more informative, sequences from both the *X. arboricola* pv. *corylina* and reference strains were used. In fact, MLSA analyses with three concatenated genes and ANI indicate that increasing the sequence length improves the ability to discriminate among bacterial isolates, highlighting the impact of environmental pressures on bacteria.

In this work, it was decided to consider the phylogenetic tree obtained using the Maximum Parsimony approach for the classification of the isolates because this method minimizes the number of nucleotide substitutions or changes and therefore can be considered robust and consistent. In this sense, it is important to highlight that the results obtained by MLSA were mostly corroborated by the analysis of complete genomes, with more than 80% of the Chilean *X. arboricola* pv. *corylina* isolates clustering in an equivalent manner. In this context, it should be noted that ANI compares whole-genome sequences (including non-essential genes), while MLSA analyzes nucleotide sequences of specific housekeeping genes. Generally, ANI provides higher resolutions for closely related isolates; therefore, it is expected to obtain a greater number of clusters. In view of the results, it was considered that both methods can be valuable for delineating species boundaries and understanding evolutionary relationships since, as corroborated in this work, their results show a correlation. Also, it is important to consider that, when using the complete genomic sequences, the effect of the geographical origin could become more evident; in this sense, it would be expected that the *X. arboricola* pv. *corylina* isolates used as references, originating from different geographical origins/countries, group differently compared to when only housekeeping genes are used.

On the other hand, the fact that four clearly distinguishable phylogenetic clades were identified in the Chilean isolates of *X. arboricola* pv. *corylina* differs from what has been reported in previous international studies, in which isolates from different geographical locations and continents were used and only two phylogenetic clades were identified when the *gyrB* and *rpoD* genes were concatenated [6,7]. In Figure 2, the reference bacterial isolates of *X. arboricola* pv. *corylina* were grouped into clades I and IV, which would indicate that there are two local phylogenetic groups in Chile. Furthermore, it is worth mentioning that, even when using only the *rpoD* and *gyrB* genes, the isolates are separated into three clearly distinguishable phylogenetic clades. It should be noted that the inclusion of the *atpD* gene provides a higher resolution among closely related isolates.

In relation to the geographic distribution of the Chilean *X. arboricola* pv. *corylina* isolates corresponding to the different phylogenetic groups, it was observed that clade I included isolates from four localities from the Maule and Araucanía Regions; clade II grouped isolates from three localities in the Ñuble and Araucanía Regions; clade III grouped isolates from four localities from the Maule, Ñuble, and Los Ríos Regions; and clade IV grouped isolates from seven localities from the Ñuble to Araucanía Regions. This last group covers the largest geographic distribution within the country. This provides a local distribution of the phylogenetic groups, showing that the isolates grouped in clade I are concentrated in a few small locations, while the isolates included in clade IV have a broader distribution toward the south of the country. It is also important to mention that three phylogenetic clades (II, III, and IV) were found in a single orchard in Coihueco, Ñuble Region. Also, two phylogenetic clades were found in a single plant from groups I and III, evidencing the relevant diversity of the *X. arboricola* pv. *corylina* isolates present in the country.

The results of the biochemical and HR characterization of the *X. arboricola* pv. *corylina* isolates coincided almost entirely with the profile reported by the EPPO [16]: negative for the oxidase test, positive for growth at 35°C, starch hydrolysis, tolerance to NaCl (5%), gelatin liquefaction, HR in the non-host plant *N. tabacum* (only for 25 of the 29 isolates of the bacteria), and utilization of glucose, trehalose, and sucrose. However, Chilean isolates also metabolize sorbitol and mannitol, which differs from what has been previously reported in strains from other regions [8,17]. These results agree with those of Lamichhane [3], reporting that Chilean isolates s obtained from the Araucanía Region metabolized D-mannitol and D-sorbitol, suggesting an adaptive metabolic capacity of *X. arboricola* pv. *corylina*, which allows them to survive and proliferate in different environmental conditions. In Chile, elevated levels of ultraviolet radiation within the wavelength range 280–320 nm (UV-B) [18] cause an increase in reactive oxygen species (ROS) which, in high concentrations, can damage plant cells. To counteract this situation, plants maintain or increase the synthesis of sorbitol and mannitol, which function as antioxidants [19]. Under these environmental conditions, plant-associated bacteria can modify their metabolism to utilize these polyols as a source of carbon. In this context, even though the biochemical properties of isolates are essential for the characterization of phytopathogenic bacteria, they are not entirely significant, so it is necessary to complement the information with molecular methods [4].

Of the four HR-negative isolates, two come from the same locality (Villarica) but belong to two different phylogenetic groups (II and IV); the third was isolated in Panguipulli and the last in Cohihueco and belong to clades IIIb and IV, respectively. Therefore, the HR-negative result would not be related to the genetic variability of these isolates. The same was observed with *P. syringae* pv. *actinidiae* from kiwifruit, but in this case the HR-positive isolates did not present differences in genomic sequences with the HR-negative ones [20]. To accurately establish the level of virulence of the HR-negative isolates s of *X. arboricola* pv. *corylina*, it is necessary to perform pathogenicity assays; however, due to the damage observed in the plant material from which they were isolated, being HR-negative should not affect their aggressiveness.

Finally, for crop health management, studying the population structure of *X. arboricola* pv. *corylina* is crucial to understanding the biological, phenotypic, and genotypic characteristics of domestic isolates and their pathogenicity. The characterization of Chilean isolates is basic to develop more effective health management strategies in orchards and promote integrated disease management to devise sustainable measures.

## 4. Materials and Methods

### 4.1. Plant Material Sampling, Bacterial Isolation, and Culture Conditions

*Xanthomonas* spp. isolates were obtained between October and November 2023 from 37 productive hazelnut fruit orchards. Unless otherwise specified, bacterial isolates were recovered from the tissue of hazelnut trees found throughout five regions belonging to the central and southern areas of Chile: Maule, Ñuble, Bío Bío, Araucanía, and Los Ríos. For bacterial isolation, plant material with disease symptoms was collected (Figure 1), such as leaves with chlorotic and necrotic spots, wilted twigs, branch and stem cankers, and also developing fruits and involucres. Details of the origin of each bacterial isolate are described in Table S1. The number of samples collected was proportional to the number of hectares; that is, one sample per 10 hectares corresponded to one plant. Of every 10 samples collected, approximately 2 were asymptomatic. To obtain the bacterial isolates, the samples were washed with running tap water and subsequently with sterile distilled water. Between 0.5 and 1 g of plant material were weighed and macerated in 5 mL of Phosphate Buffered Saline (PBS; 137 mM NaCl, 2.7 mM KCl, 10 mM Na_2_HPO_4_, and 1.8 mM KH_2_PO_4;_ pH: 7.4). Three dilutions of the macerate were prepared using PBS: 1/10, 1/100, and 1/1000. Bacterial isolation was conducted under sterile conditions by inoculating 200 µL of each of these dilutions onto solid King’s B medium (KB) (2% peptone, 0.15% K_2_PO_4_, 0.15% MgSO_2_ × 7H_2_O, 1.5% glycerol, and 1.5% agar; pH 7). The plates were incubated at 27°C for 72 h. The initial selection of bacterial isolates was conducted based on the macroscopic characteristics of the colonies, such as color, shape, brightness, and mucosity. Then, the genus of each bacterium isolated was confirmed by sequencing the 16S rRNA gene and comparing with the sequences available in the GenBank database. The isolates were cultured in solid KB medium and incubated at 27°C for 72 h. For long-term maintenance, the isolates were cryopreserved and stored at −80°C in nutrient broth (NB; meat extract 0.3% and peptone 0.5%) with 15% glycerol.

### 4.2. Molecular Characterization of Bacterial Isolates

The genomic DNA of bacterial isolates was extracted by using the Presto Mini gDNA Bacteria Kit (Geneaid Biotech, New Taipei City, Taiwan, China) according to the manufacturer’s instructions. PCR amplification of the 16S rRNA, *rpoD*, and *gyrB* genes was conducted by using the primer pairs 16S 27- F +16S 1492- R, rpoD- SOF4+rpoDx- SoR6, and gyrB1F+gyrB1R, respectively (Table 1).

PCR reactions were conducted in 30 µL containing 20.8 µL of H_2_O, 3 µL of PCR buffer (10×), 1.5 µL of MgCl_2_ (50 µM), 1 µL of dNTPs (10 µM), 1 µL of each primer (0.75 µL of H_2_O and 0.25 µL of primer), 0.2 µL of Taq polymerase, and 1.5 µL of DNA. Amplification conditions for the 16S rRNA fragment consisted of initial denaturation for 4 min at 96°C, followed by thirty cycles of 30 s at 94°C, 30 s at 57°C, and 1 min at 72°C, plus a final extension of 10 min at 72°C. Meanwhile, amplification conditions for the *rpoD* gene consisted of initial denaturation for 3 min at 95°C, followed by thirty-five cycles of 30 s at 95°C, 1 min at 58°C, and 45 s at 72°C, and a final extension of 5 min at 72°C. For *gyrB* gene, amplification conditions consisted of initial denaturation for 5 min at 95°C, followed by thirty-five cycles of 30 s at 95°C, 45 s at 56°C, and 1 min at 72°C, plus a final extension of 7 min at 72°C.

The amplification products were analyzed by 1.2% agarose with ethidium bromide gel electrophoresis using 1X TAE buffer (40 mM Tris, 20 mM acetic acid, and 1 mM ETDA; pH 8.0). Electrophoresis was run at 145 V for 35 min. The amplification products were visualized using a UV transilluminator. Subsequently, the amplicons of the 29 bacterial isolates were purified and sequenced by an external service at Psomagen (Rockville, MD, USA).

Single-gene nucleotide sequences were edited, assembled, aligned, trimmed, and compiled by using Bioedit Sequence Alignment Editor v.7.7.1 software [23].

To verify the genetic variability of the 29 isolates of *X. arboricola* pv. *corylina*, three *loci* corresponding to *rpoD*, *gyrB*, and *atpD* housekeeping genes were used for MLSA analysis. The *atpD* gene sequence of each isolate was obtained by sequencing the whole genomes using the DNBSEQ-G400 platform (MGI-Tech, San José, CA, USA), with library preparation carried out using the MGIEasy FS DNA Library Prep Set. Raw reads were quality-trimmed and assembled using CLC Genomics Workbench v24.0.1 software. The resulting genome assemblies were compared using the Whole Genome Alignment module within the same software, employing ANI and phylogenetic tree construction to assess genomic similarity and evolutionary relationships among isolates.

As reference for the whole-genome sequences, isolates of *X. arboricola* pv. *corylina* and the pathovars *pruni* and *juglandis,* available in the GenBank database, were included (Table 2). These sequences were downloaded and aligned with the sequences obtained in this study. For the MLSA, the gene sequences were concatenated and aligned, ending in a sequence of 1660 bp or 3032 bp when using two (*rpoD* and *gyrB*) or three (*rpoD*, *gyrB,* and *atpD*) genes, respectively. Finally, for genetic relationship analyses, neighbor-joining (NJ) and Maximum Parsimony trees were generated in MEGA 7.0.26 software [24].

### 4.3. Biochemical Characterization

The biochemical characterization of the *X. arboricola* pv. *corylina* isolates was performed following the protocols described by Schaad et al. [25]. The tests were based on the following: growth at 35°C, NaCl tolerance (5%), oxidase reaction, starch hydrolysis, gelatin liquefaction, and utilization sources of the following carbohydrates: glucose, sucrose, trehalose, mannitol, and sorbitol. For the growth tests at 35°C, NaCl tolerance (5%), starch hydrolysis, and utilization sources of all the aforementioned carbohydrates, the bacteria were grown overnight in 3 mL of Luria–Bertani (LB) [26] medium with shaking. The following day, 30 µL were diluted in 3 mL of LB medium and allowed to grow until an absorbance of 0.1, equivalent to 1 × 10^8^ CFU/mL, was reached. Aliquots of 5 µL were used for growth tests on Petri dishes, with 3 repetitions per isolate.

### 4.4. Hypersensitivity Response (HR)

To determine the pathogenic potential of the *X. arboricola* pv. *corylina* isolates, HR assay was performed on leaves of 60-day-old plants of *Nicotiana tabacum* L. cultivar White Burley [6,25]. Aliquots of 30 µL from overnight cultures of the bacterial isolates were used to inoculate 3 mL of LB liquid medium, and the bacteria were allowed to grow until they reached an absorbance of 0.1, equivalent to 1 × 10^8^ CFU/mL [6]. Then, 0.5 mL was infiltrated into the mesophyll of the upper part of the tobacco leaves. Two replicates were performed for each isolate. Infiltrations were also performed with *Pantoea agglomerans* as a negative control, *P. syringae* pv. *actinidiae* as a positive control, and NB as a negative control. Each isolate and control were infiltrated into different leaves. Photographic registers were taken 48 h post-infiltration.

## Figures and Tables

**Table 1:** Primers used in this study for amplification of 16S rRNA, *rpoD,* and *gyrB* genes.

Primer	Sequence (5′–3′)	Amplicon (bp)	Reference
16S 1492-R	TACGGCTACCTTGTTACGACTT	1470	[21]
16S 27-F	AGAGTTTGATCCTGGCTCAG		
rpoD-SOF4	GGAGCAGATCGAAGACATCATCAG	951	[22]
rpoDx-SoR6	CATCTCGATCGAGCCCTG		
gyrB1R	CCCATCARGGTGCTGAAGAT	904	[7]
gyrB1F	ACGAGTACAACCCGGACAA		

**Table 2:** Details of bacterial isolates used as references for MLSA analysis.

Bacteria Pathovars	Isolate Acronyms	Country of Origin	GenBank Accession Number
*Xantomonas arboricola* pv. *corylina*	Xac 301	Poland	HG992338
	Xac CFBP 1846	France	CP076619
	Xac CFBP 2565	France	NZ_MDSJ00000000
	Xac CFBP 1159	USA	NZ_MDEA00000000
	Xac A7	Chile	CP062164
*Xantomonas arboricola* pv. *juglandis*	Xaj 417	USA	CP012251
	OS4	Serbia	NZ_JASVYK000000000
*Xantomonas arboricola* pv. *pruni*	Xap Xcp1	USA	CP090954
	Xap T1	USA	CP091075

## Data Availability

All data are available in the manuscript and in Supplementary Materials. Sequences can be accessed via GenBank accession numbers.

## Supplementary Materials

The following supporting information can be downloaded at https://www.mdpi.com/article/10.3390/plants14203148/s1, Figure S1: bacterial colonies with *X. arboricola* pv. *corylina*-like morphology in KB medium; Figure S2: geographic locations of the phylogenetic groups indicated by color (phylogenetic group I: yellow; II: orange; III: green; IV: blue); Figure S3: biochemical tests performed on *X. arboricola* pv. *corylina* isolates: (a) growth at 35°C; (b) starch hydrolysis; (c) growth in medium with sucrose used as a carbon source; (d) growth in medium with mannitol; (e) growth in medium with sorbitol; (f) growth in medium with glucose; (g) growth in medium with trehalose; Figure S4: hypersensitivity response reaction at 48 h after inoculation: (a) HR induced by the isolate AGR56; (b) chlorosis induced by the isolate 157o; (c) negative control *Pantoea agglomerans;* (d) positive HR response induced by *Pseudomonas syringae* pv. *actinidiae*; Figure S5: Heat map of the average nucleotide identity (ANI) for the 29 *X. arboricola* pv. *corylina* isolates; Figure S6: phylogenetic tree using the neighbor-joining method obtained by concatenating the *rpoD* and gryB genes. Evolutionary distances were calculated using the Composite Maximum Likelihood method. Each color indicates a distinct *X. arboricola* pv. *corylina* phylogenetic group. The red triangles indicate the *X. arboricola* pv. *corylina* reference strains. The nucleotide sequences obtained for each gene from the different isolates were aligned and trimmed, yielding sequences of 880 and 780 bp in length, for the *rpoD* and *gyrB* genes, respectively. Gene sequences were aligned and concatenated, ending in a sequence of 1660 bp; Table S1: *X. arboricola* pv. *corylina* isolates used in this work; Table S2: reference isolates used in this work; Table S3: biochemical tests of *X. arboricola* pv. *corylina* isolates; Table S4: use of different carbon sources by the different *X. arboricola* pv. *corylina* isolates; Table S5: hypersensitivity response (HR) in *N. tabacum* of *X. arboricola* pv. *corylina* isolates.
