# Supplementary material for: Molecular and Biochemical Characterization of Xanthomonas arboricola pv. corylina Isolates Infecting Hazelnut Orchards in Chile
Source: Plants (Basel). 2025 Oct 13;14(20):3148. doi: 10.3390/plants14203148 (PMC12567208; doi:10.3390/plants14203148)
Supplement: Supplementary file 1 [file plants-14-03148-s001.zip › plants-3838978-supplementary.pdf]

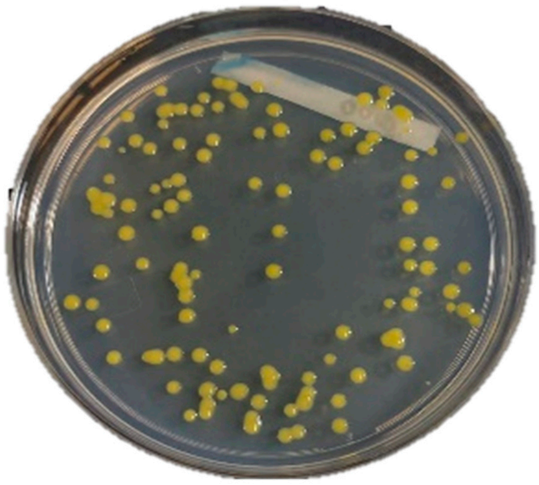

Figure S1. Bacterial colonies with *X. arboricola* pv. *corylina*-like morphology in KB medium.

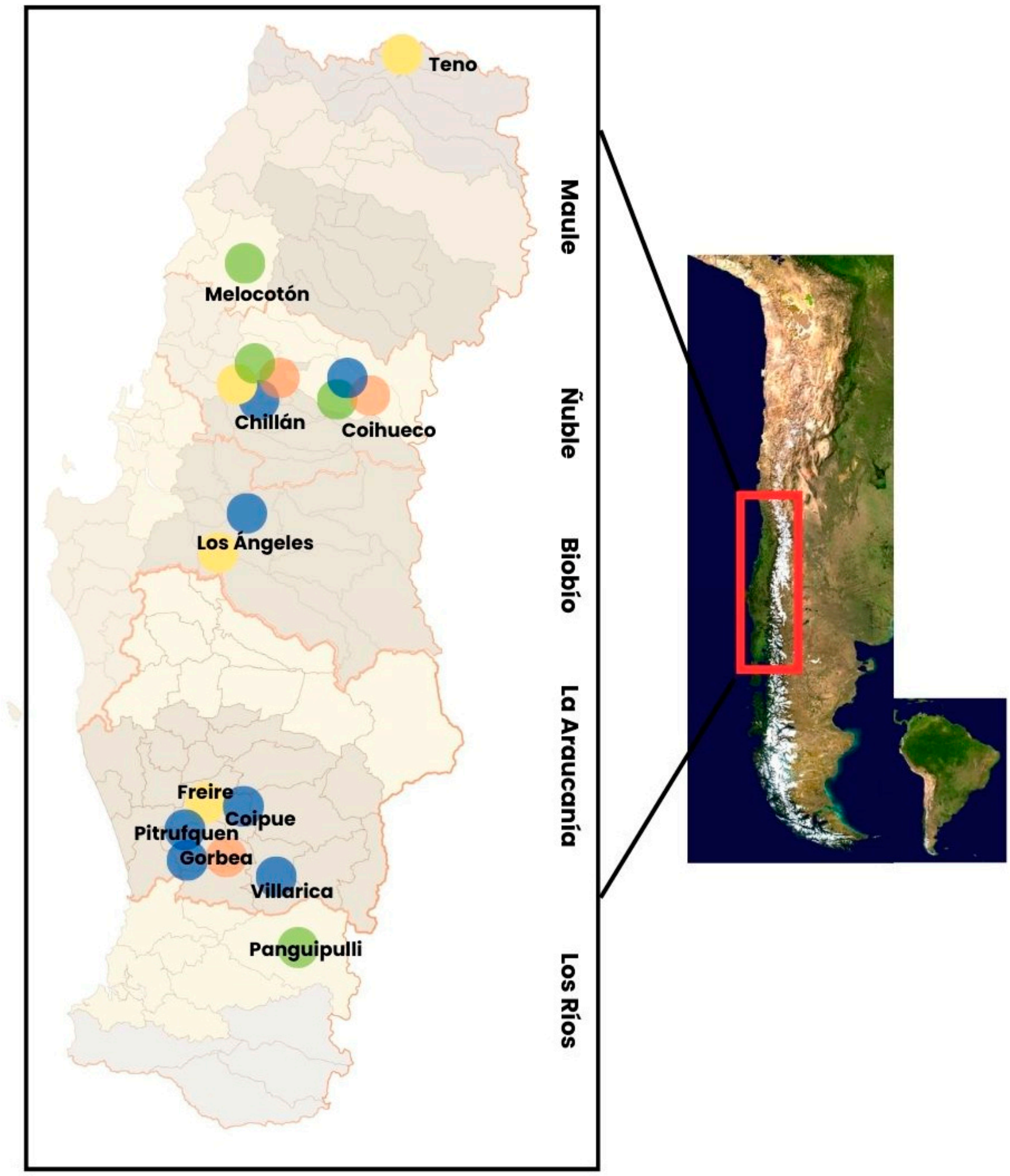

Figure S2. Geographic location of the *X. arboricola* pv. *corylina* phylogenetic groups indicated by color (group I: yellow; II: orange; III: green; IV: blue).

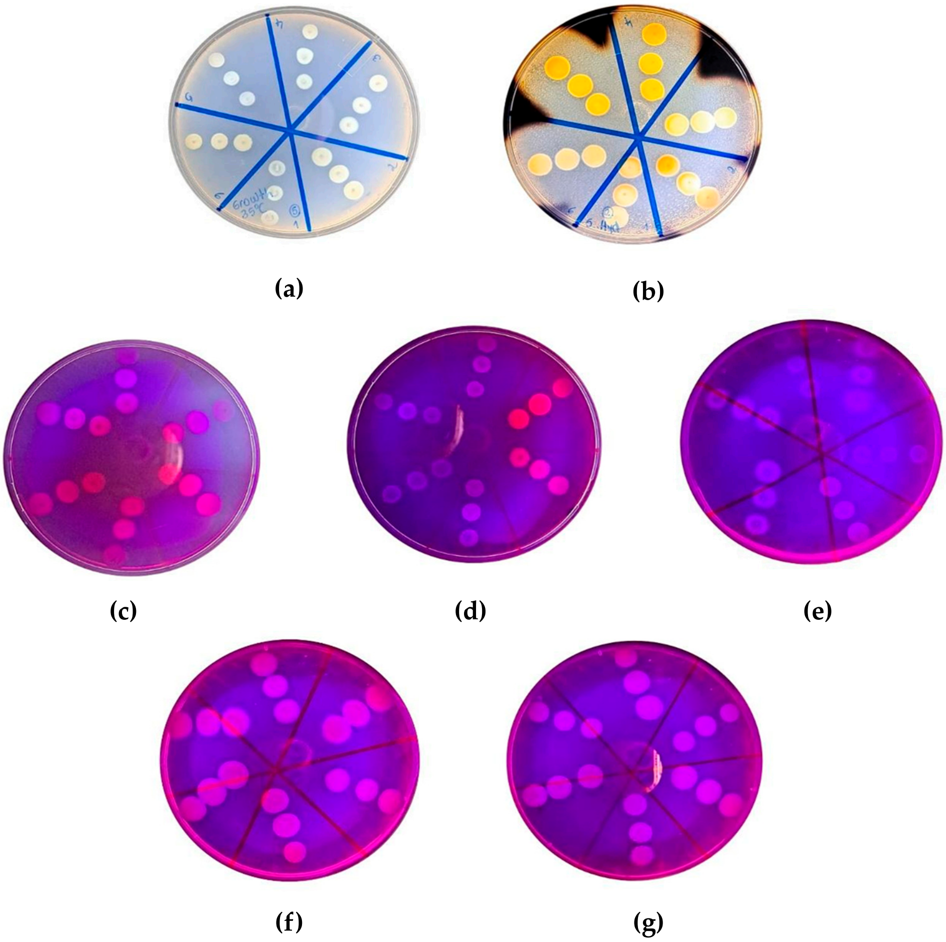

(a) (b) (c) (d) (e) (f) (g)

Figure S3. Biochemical tests performed on *X. arboricola* pv. *corylina* isolates: a) growth at 35°C, b) starch hydrolysis, c) growth in medium with sucrose used as a carbon source, d) growth in medium with mannitol, e) growth in medium with sorbitol, f) growth in medium with glucose, g) growth in medium with trehalose.

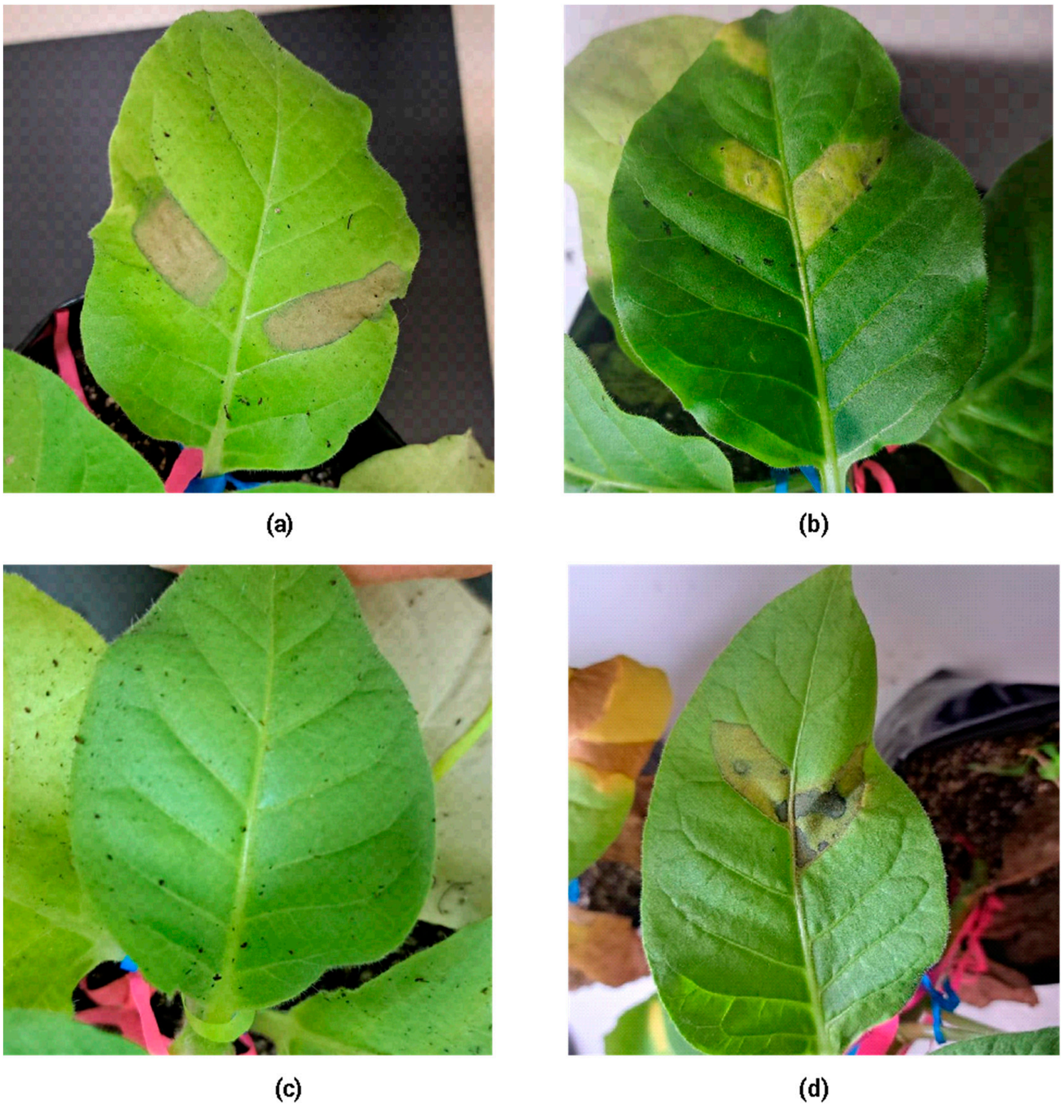

Figure S4. Hypersensitivity response (HR) reaction at 48 hours after inoculation: a) HR corresponding to *X. arboricola* pv. *corylina* isolate AGR56; b) chlorosis corresponding to *X. arboricola* pv. *corylina* isolate 157o; c) negative control *Pantoea agglomerans*; d) positive HR response induced by *Pseudomonas syringae* pv. *actinidiae*.

Table S1. Chilean *Xanthomonas arboricola* pv. *corylina* strains used in this work.

Isolate	Hazelnut variety	Symptoms[1]	Region	Location	GenBank Accession Numbers			
					gyrB	*rpoD*	Biosample	Genome
**OF346-HA2***	Tonda di Giffoni	AL	Maule	Teno	PX273309	PX273348	SAMN51001177	JBQWSD000000000
**OF348-HC4***	Tonda di Giffoni	CL	Ñuble	Chillán	PX273310	PX273349	SAMN51001178	JBQWSC000000000
**OF349-HN4***	Tonda di Giffoni	NL	Ñuble	Chillán	PX273314	PX273353	SAMN51001181	JBQWRZ000000000
**OF349-FN3***	Tonda di Giffoni	NP	Ñuble	Chillán	PX273312	PX273351	SAMN51001179	JBQWSB000000000
**OF349-HC8***	Tonda di Giffoni	CL	Ñuble	Chillán	PX273313	PX273352	SAMN51001180	JBQWSA000000000
**OF351-HN1***	Tonda di Giffoni	NL	Ñuble	Chillán	PX273315	PX273354	SAMN51437148	JBRBCR000000000
**OF355-FN2***	Tonda di Giffoni	NP	Ñuble	Chillán	PX273317	PX273356	SAMN51001182	JBQWRY000000000
**OF356-HN7***	Tonda di Giffoni	NL	Ñuble	Chillán	PX273319	PX273358	SAMN51001183	JBQWRX000000000
**OF360-HA8***	Barcelona	AL	Ñuble	Coihueco	PX273323	PX273362	SAMN51001184	JBQWRW000000000
**OF361-HA4***	Barcelona	AL	Ñuble	Coihueco	PX273324	PX273363	SAMN51001185	JBQWRV000000000
**OF362-HN1***	Barcelona	NL	Ñuble	Coihueco	PX273325	PX273364	SAMN51001186	JBQWRU000000000
**OF364-HA7***	Barcelona	AL	Ñuble	Coihueco	PX273326	PX273365	SAMN51001187	JBQWRT000000000
**OF365-HA4***	Tonda di Giffoni	AL	Ñuble	Coihueco	PX273327	PX273366	SAMN51001188	JBQWRS000000000
**OF373 -HN3***	Barcelona	NL	Araucanía	Freire	PX273328	PX273367	SAMN51001189	JBQWRR000000000
**OF379 -HN5***	Tonda di Giffoni	NL	Araucanía	Coipue	PX273330	PX273369	SAMN51001190	JBQWRQ000000000
**OF381 -I4***	Tonda di Giffoni	I	Biobío	Los Ángeles	PX273332	PX273371	SAMN51001192	JBQWRO000000000

**OF381 -HC7***	Tonda di Giffoni	CL	Biobío	Los Ángeles	PX273331	PX273370	SAMN51001191	JBQWRP000000000
**OF383-I4***	Barcelona	I	Maule	Melocotón	PX273333	PX273372	SAMN51001193	JBQWRN000000000
**128o****	Tonda di Giffoni	NP	Araucanía	Villarrica	PX273295	PX273334	SAMN51008458	In progress
**132o****	Unknown	NL	Biobío	Los Ángeles	PX273296	PX273335	SAMN51001169	JBQWSL000000000
**136o****	Barcelona	NL	Ñuble	Chillán	PX273297	PX273336	SAMN51008459	In progress
**140o****	Barcelona	NL	Ñuble	Chillán	PX273298	PX273337	SAMN51008460	In progress
**142o****	Tonda di Giffoni	NL	Araucanía	Gorbea	PX273300	PX273339	SAMN51001170	JBQWSK000000000
**143o****	Unknown	NL	Araucanía	Gorbea	PX273301	PX273340	SAMN51001171	JBQWSJ000000000
**144o****	Barcelona	NL	Araucanía	Pitrufquén	PX273302	PX273341	SAMN51001172	JBQWSI000000000
**157o****	Barcelona	NL	Araucanía	Panguipulli	PX273304	PX273343	SAMN51001173	JBQWSH000000000
**AGR 55*****	Unknown	n.i.	n.i.	n.i.	PX273306	PX273345	SAMN51001174	JBQWSG000000000
**AGR 56*****	Unknown	n.i.	n.i.	n.i.	PX273307	PX273346	SAMN51001175	JBQWSF000000000
**IA Ufro****	Tonda di Giffoni	NP	Araucanía	Cunco	PX273308	PX273347	SAMN51001176	JBQWSE000000000

[1]NL: leaves with necrotic spots; CL: leaves with chlorotic spots; NP: necrotic phloem; AL: asymptomatic leaves; I: involucres; n.i.: no information. Collections of bacterial strains: *University of Chile; **University of O'Higgins; ***Estación Experimental Agroñuble Research, Chillán, Chile. The sequences are deposited under Bioproject PRJNA1314811.

Table S2. Reference strains used in this work.

Isolate	*X. arboricola* pv.	Plant species	Country	Acc. Number
**Xap_33-1.0**	*pruni*	*Prunus dulcis L.*	Spain	JHUQ00000000
**Xap_CITA_9**	*pruni*	*Prunus persica L.*	Spain	RWYS00000000
**Xap T1**	*pruni*	*Prunus persica L.*	USA	CP091075
**Xap Xcp1**	*pruni*	*Prunus persica var. nucipersica*	USA	CP090954
**Xaj CFBP_427**	*juglandis*	*Juglans regia* L.	Portugal	UNRO00000000
**Xaj OS4**	*juglandis*	*Juglans regia* L.	Serbia	JASVYK000000000
**Xaj CFBP 765**	*juglandis*	*C. illinoinensis*	Portugal	HG999365
**Xaj 417**	*juglandis*	*Juglans regia* L.	USA	CP012251
**A7_Cl**	*corylina*	*Corylus avellana* L.	Chile	CP062164
**Xac_301**	*corylina*	*Corylus avellana* L.	Switzerland	HG992338
**Xac CFBP 2565**	*corylina*	*Corylus avellana* L.	France	NZ_MDSJ00000000
**Xac CFBP 1159**	*corylina*	*Corylus avellana* L.	Switzerland	NZ_MDEA00000000
**Xac CFBP 1846**	*corylina*	*Corylus avellana* L.	France	CP076619

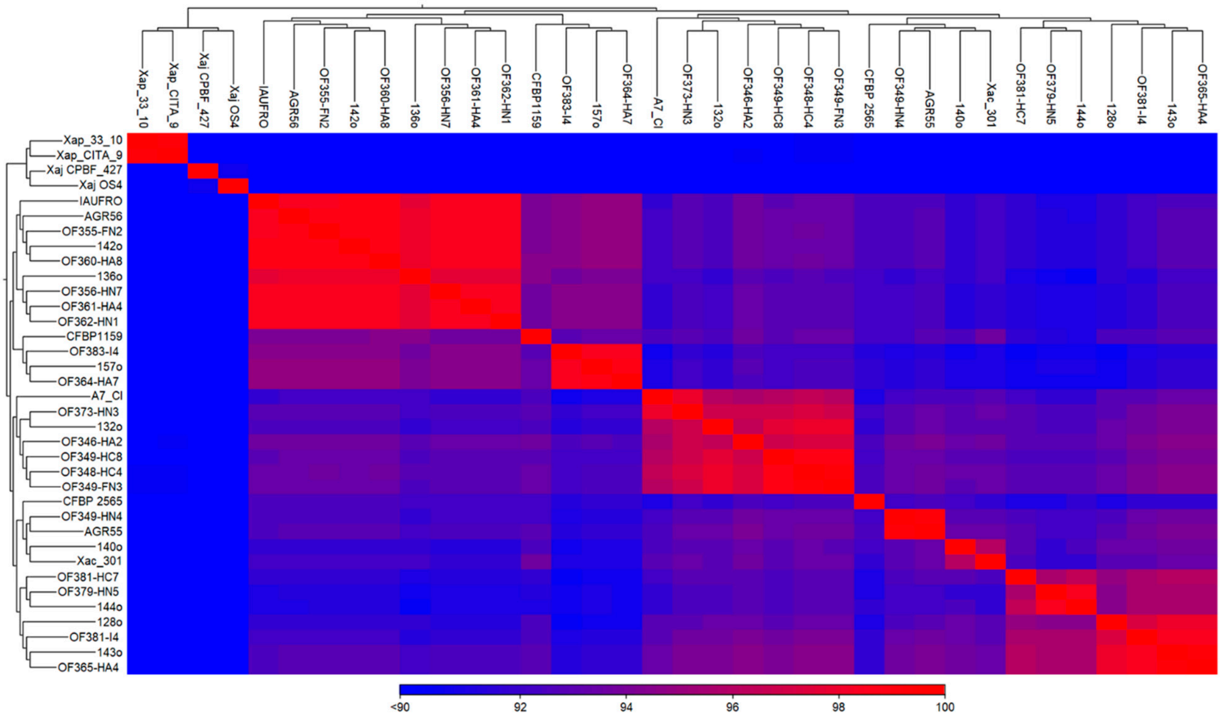

Figure S5. Heat map of the average nucleotide identity (ANI) for the 29 *X. arboricola* pv. *corylina* isolates from this study (Table S1). Chilean and reference strains GenBank accession numbers are in tables S1 and S2.

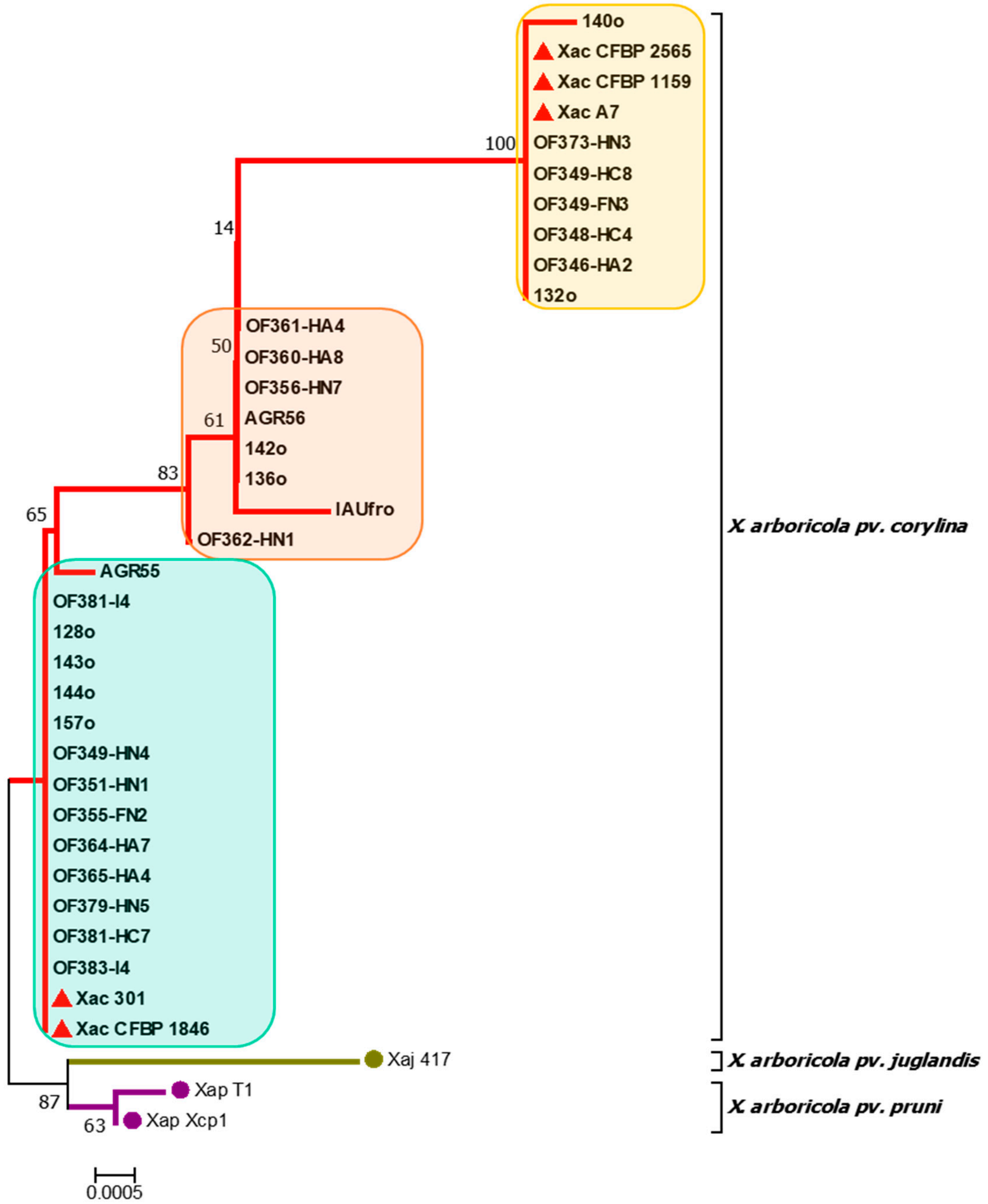

Figure S6. Phylogenetic tree using the Neighbor-Joining method obtained by concatenating the *rpoD* and *gyrB* genes. Evolutionary distances were calculated using the Composite Maximum Likelihood method. Each color indicates a distinct *X. arboricola* pv. *corylina* phylogenetic group. The red triangles indicate the *X. arboricola* pv. *corylina* reference isolates. The nucleotide sequences obtained for each gene from the different isolates were aligned and trimmed, yielding sequences of 880 and 780 bp in length, for the *rpoD* and *gyrB* genes, respectively. Gene sequences were aligned and concatenated ending in a sequence of 1660 bp. Chilean and reference strains GenBank accession numbers are in tables S1 and S2.

Table S3. Biochemical tests of *X. arboricola* pv. *corylina* isolates.

Isolate	Phylogenetic group	Growth at 35°C	Starch hydrolysis	NaCl (5%) tolerance	Liquefaction of gelatin	Oxidase reaction
132o	I	+	+	++	+	-
OF373-HN3	I	+	+	+	+	-
OF349-HC8	I	+	+	+++	+	-
OF349-FN3	I	+	+	+	+	-
OF348-HC4	I	+	+	++	+	-
OF346-HA2	I	+	+	++	+	-
140o	I	+	+	+	+	-
142o	II	+	+	++	+	-
AGR56	II	+	+	+	+	-
IAUfro	II	+	+	++	+	-
OF356-HN7	II	+	+	+	+	-
OF360-HA8	II	+	+	+	+	-
136o	II	+	+	++	+	-
OF361-HA4	II	+	+	+	+	-
OF362-HN1	II	+	+	++++	+	-
AGR55	III	+	+	+-	+	-
OF349-HN4	III	+	+	+	+	-
OF355-FN2	III	+	+	++	+	-
OF364-HA7	III	+	+	++++	+	-
157o	III	+	+	+	+	-
OF383-I4	III	+	+	++	+	-
128o	IV	+	+	+	+	-
144o	IV	+	+	+	+	-
OF351-HN1	IV	+	+	+++	+	-
OF365-HA4	IV	+	+	+	+	-
OF379-HN5	IV	+	+	++	+	-
OF381-HC7	IV	+	+	+	+	-
OF381-I4	IV	+	+	++	+	-
143o	IV	+	+	+	+	-

Table S4. Use of different carbon sources by the different *X. arboricola* pv. *corylina* isolates.

Isolate	Phylogenetic group	Sucrose	Mannitol	Sorbitol	Glucose	Trehalose
132o	I	+	+	++	+	+
OF373-HN3	I	+	+	+	+	+
OF349-HC8	I	+	+	+++	+	+
OF349-FN3	I	+	+	+	+	+
OF348-HC4	I	+	+	+	+	+
OF346-HA2	I	+	+	+	+	+
140o	I	+	+	++	+	+
142o	II	+	+	+	+	+
AGR56	II	+	+	++	+	+
IAUfro	II	+	+	++	+	+
OF356-HN7	II	+	+	+	+	+
OF360-HA8	II	+	+	+	+	+
136o	II	+	+	++	+	+
OF361-HA4	II	+	+	+	+	+
OF362-HN1	II	+	+	+	+	+
AGR55	III	+	+	+	+	+
OF349-HN4	III	+	+	+	+	+
OF355-FN2	III	+	+	+	+	+
OF364-HA7	III	+	+	+++	+	+
157o	III	+	+	+	+	+
OF383-I4	III	+	+	++	+	+
128o	IV	+	+	+	+	+
144o	IV	+	+	++	+	+
OF351-HN1	IV	+	+	++	+	+
OF365-HA4	IV	+	+	+	+	+
OF379-HN5	IV	+	+	+	+	+
OF381-HC7	IV	+	+	++	+	+
OF381-I4	IV	+	+	++	+	+
143o	IV	+	+	+	+	+

Table S5. Hypersensitivity response (HR) in *N. tabacum* of *X. arboricola* pv. *corylina* isolates.

**Isolate**	**Phylogenetic group**	**HR**
132o	I	+
OF373-HN3	I	+
OF349-HC8	I	+
OF349-FN3	I	+
OF348-HC4	I	+
OF346-HA2	I	+
140o	I	+
142o	II	chlorosis
AGR56	II	+
IAUfro	II	+
OF356-HN7	II	+
OF360-HA8	II	+
136o	II	+
OF361-HA4	II	+
OF362-HN1	II	+
AGR55	III	+
OF349-HN4	III	+
OF355-FN2	III	+
OF364-HA7	III	+
157o	III	chlorosis
OF383-I4	III	+
128o	IV	chlorosis
144o	IV	+
OF351-HN1	IV	+
OF365-HA4	IV	chlorosis
OF379-HN5	IV	+
OF381-HC7	IV	+
OF381-I4	IV	+
143o	IV	+
